# Corrosion Behavior of Ni-Cr Alloys with Different Cr Contents in NaCl-KCl-MgCl_2_

**DOI:** 10.3390/ma17102335

**Published:** 2024-05-14

**Authors:** Peng Lei, Lizhen Zhou, Yu Zhang, Fuli Wang, Qinzhe Li, Jiangyan Liu, Xueyun Xiang, Hang Wu, Wen Wang, Fuhui Wang

**Affiliations:** 1 Shenyang National Laboratory for Materials Science, Northeastern University, Shenyang 110819, China; leipeng1999106@163.com (P.L.); wszb157@163.com (Y.Z.); wangfuli1122@163.com (F.W.); 13205489190@163.com (Q.L.); ljy081722@163.com (J.L.); xiangxueyun1998@163.com (X.X.); wen@imr.ac.cn (W.W.); fhwang@mail.neu.edu.cn (F.W.); 2 School of Materials Science and Engineering, Northeastern University, Shenyang 110819, China; 3 Institute of Metals, Chinese Academy of Sciences, Shenyang 110016, China

**Keywords:** Ni-Cr alloy, NaCl-KCl-MgCl_2_ molten salt, corrosion behavior, corrosion mechanism

## Abstract

This study investigates the corrosion behavior of Ni-Cr binary alloys, including Ni-10Cr, Ni-15Cr, Ni-20Cr, Ni-25Cr, and Ni-30Cr, in a NaCl-KCl-MgCl_2_ molten salt mixture through gravimetric analysis. Corrosion tests were conducted at 700 °C, with the maximum immersion time reaching up to 100 h. The corrosion rate was determined by measuring the mass loss of the specimens at various time intervals. Verifying corrosion rates by combining mass loss results with the determination of element dissolution in molten salts using Inductively Coupled Plasma Optical Emission Spectroscopy (ICP-OES). Detailed examinations of the corrosion products and morphology were conducted using X-ray diffraction (XRD) and scanning electron microscopy (SEM). Micro-area elemental analysis on the corroded surfaces was performed using an energy dispersive spectrometer (EDS), and the elemental distribution across the corrosion cross-sections was mapped. The results indicate that alloys with lower Cr content exhibit superior corrosion resistance in the NaCl-KCl-MgCl_2_ molten salt under an argon atmosphere compared to those with higher Cr content; no corrosion products were retained on the surfaces of the lower Cr alloys (Ni-10Cr, Ni-15Cr). For the higher Cr alloys (Ni-20Cr, Ni-25Cr, Ni-30Cr), after 20 h of corrosion, a protective layer was observed in certain areas. The formation of a stable Cr_2_O_3_ layer in the initial stages of corrosion for high-Cr content alloys, which reacts with MgO in the molten salt to form a stable MgCr_2_O_4_ spinel structure, provides additional protection for the alloys. However, over time, even under argon protection, the MgCr_2_O_4_ protective layer gradually degrades due to chloride ion infiltration and chemical reactions at high temperatures. Further analysis revealed that chloride ions play a pivotal role in the corrosion process, not only facilitating the destruction of the Cr_2_O_3_ layer on the alloy surfaces but also possibly accelerating the corrosion of the metallic matrix through electrochemical reactions. In conclusion, the corrosion behavior of Ni-Cr alloys in the NaCl-KCl-MgCl_2_ molten salt environment is influenced by a combination of factors, including Cr content, chloride ion activity, and the formation and degradation of protective layers. This study not only provides new insights into the corrosion resistance of Ni-Cr alloys in high-temperature molten salt environments but also offers significant theoretical support for the design and optimization of corrosion-resistant alloy materials.

## 1. Introduction

Concentrated Solar Power (CSP) represents an emerging technology for sustainable electricity generation [1], harnessing sunlight concentrated to heat a thermal transfer fluid (HTF), which is then pumped to a heat exchanger to produce steam that drives turbines for electricity generation. The HTF is one of the most crucial components for the overall performance and efficiency of CSP systems. Current CSP plants utilize nitrate-based molten salts as the HTF, capable of operating over several hours of energy storage. The aim for next-generation solar energy conversion systems in CSP applications is to operate with advanced fluids at high temperatures ranging from 600–800 °C, surpassing the stability temperatures of nitrates. At the higher operational temperatures achievable in solar tower CSP plants, chloride salts, due to their thermal stability and generally lower costs, are considered ideal candidates for thermal energy storage materials [2]. Ionic metal chloride salts, such as NaCl, CaCl_2_, MgCl_2_, and KCl, are abundant in nature and boil at temperatures above 1400 °C, emerging as potential high-temperature HTFs [3]. Eutectic systems of molten chloride salts exhibit advantages such as low cost, wide operational temperature ranges for thermal storage, supercooling heat, low viscosity, and good thermal stability. However, molten chloride salts can aggressively corrode containers and pipeline alloys within CSP systems [4]. Corrosion remains a major concern, especially under high-temperature operational conditions [2].

Nickel-based alloys have been selected as candidate materials for the next generation of CSP due to their excellent high-temperature strength and corrosion resistance [5,6,7]. For alloys with the same matrix element, the extent of corrosion significantly depends on the content of alloying elements [8]. Extensive experimental studies by the Oak Ridge National Laboratory have identified selective corrosion of the Cr element in nickel-based high-temperature alloys as the primary corrosion mechanism in molten salts. This process might not be readily apparent in the short term but, over the long term, is predominantly controlled by the diffusion rate of Cr elements within the alloy. However, the focus on the effect of the Cr element on corrosion has led to some ambiguous conclusions [8,9,10]. For instance, Williams compared the corrosion results in chloride salts at Brookhaven National Laboratory and found that the effect of Cr content in the alloy seemed not to be a critical factor [10]. In contrast, further research by Olson et al. [11] demonstrated a close relationship between the alloy’s molten salt corrosion resistance and its Cr content, with an increase in Cr content exacerbating alloy corrosion.

Therefore, assessing the corrosion resistance of nickel-based alloys with the same matrix elements in molten chloride salts remains challenging based on published results. To deeply understand the corrosion mechanisms of nickel-based alloys in molten salts, we selected Ni-Cr binary alloys as our research subjects, aiming to observe the impact of varying Cr content on corrosion behavior. Five different Cr-content Ni-Cr binary model alloys were prepared using a vacuum arc melting furnace. These alloys were then subjected to a series of static corrosion tests in NaCl-KCl-MgCl_2_ molten salts under various temperatures and durations to simulate the extreme environments encountered in CSP systems. The corroded alloy samples were thoroughly analyzed using a combination of advanced material characterization techniques, including weight loss measurement, Inductively Coupled Plasma Optical Emission Spectroscopy (ICP-OES), Scanning Electron Microscopy (SEM), Energy Dispersive Spectroscopy (EDS), and X-ray Diffraction. By integrating these advanced characterization techniques, we can not only reveal changes in the microstructure of the alloys but also understand in detail how Cr content influences the corrosion behavior and mechanisms of Ni-Cr binary alloys in molten salts. This provides vital experimental data and a theoretical basis for designing efficient and corrosion-resistant nickel-based alloys.

## 2. Experimental

### 2.1. Materials

The NaKMgCl molten salt system used in this study consists of a eutectic mixture of NaCl, KCl, and MgCl_2_, melted together in specific proportions. The molar ratio of these chloride salts is NaCl:KCl:MgCl_2_ = 27.5:32.5:40 [12]. Due to the significant impact of impurities in the molten salt on material corrosion, it was necessary to purify the salts. The three types of molten salts were mixed according to the specified ratios and poured into an alumina crucible. This crucible was then placed into a vertical tube furnace. A vacuum pump was used to evacuate the furnace to achieve a vacuum level of −0.1 MPa (absolute vacuum) to check the furnace’s seal integrity. After confirming that the seal met experimental standards, high-purity argon gas was introduced until the vacuum pressure gauge returned to 0 MPa. This vacuuming and argon gas-filling process was repeated 2–4 times to ensure the complete removal of residual air from the furnace. Subsequently, high-purity argon gas was slowly fed into the furnace at a rate of approximately 200 mL/min, with the exhaust valve of the tube furnace open to maintain stable pressure. At this point, the furnace atmosphere was covered with high-purity argon gas. The temperature inside the furnace was then increased to 300 °C and maintained for 24 h to remove any residual moisture from the molten salts before they reached the melting point. The temperature was then raised to 600 °C to melt the salts and maintained for 10 h to expel as much residual O_2_, H_2_O, and other impurities as possible. After this process, the furnace was allowed to cool to room temperature. The prepared ternary NaCl-KCl-MgCl_2_ molten salt was extracted from the crucible and stored in a glove box filled with high-purity argon gas to prevent the molten salt from absorbing water and oxygen from the air [13].

In this study, the ternary NaCl-KCl-MgCl_2_ molten salt has a melting point of 383 °C. Its primary physicochemical properties at 700 °C are summarized in Table 1 [12].

The alloys used in this experiment were procured from Beijing Tangshan Haoba Target Technology Co., Ltd. (Tangshan, Hebei, China). The composition of these alloys is presented in Table 2. Initially, alloys with five different Cr contents were melted into square plates measuring 100 mm × 100 mm × 2 mm. Each plate was then sectioned into smaller pieces of 10 mm × 10 mm × 2 mm using an electric discharge wire cutting machine. Subsequently, these samples were sequentially sanded with 150#, 400#, 800#, and 1200# SiC abrasive paper, followed by polishing with 0.5 μm alumina polishing paste. After polishing, the samples were cleaned for 10 min in anhydrous ethanol and deionized water using a high-frequency CNC ultrasonic cleaner and dried with a blower. Post-drying, the samples were precisely measured for length, width, and height using calipers, and their surface area was calculated. The initial mass (*m*_0_) of the samples before corrosion was recorded using an analytical balance. All samples were sealed in zip-lock bags and stored in an argon-filled glove box awaiting further experimentation [14,15,16].

### 2.2. Experimental Methods

To ensure the reliability and statistical significance of the experimental results, two sets of parallel experiments were conducted for each chromium-content alloy sample, with each set consisting of three independent specimens (*n* = 3). One set, designated as Group A, was used for weighing and calculating the average mass change before and after corrosion. The other set, designated as Group B, was used for characterization tests including SEM, EDS, and XRD. This duplication is intended to evaluate data consistency and reduce the impact of random errors.

The three specimens of each alloy were horizontally placed in two separate alumina crucibles to ensure a consistent contact surface with the molten salt, and 50 g of NaCl-KCl-MgCl_2_ solid salt mixture was added to each Al_2_O_3_ crucible (all procedures were conducted in an argon-filled glove box). This setup ensures uniformity of experimental conditions and reproducibility of results. Data processing and analysis for each experiment will be based on the results from six independent samples, further enhancing the credibility of the research conclusions.

**Figure 1:**
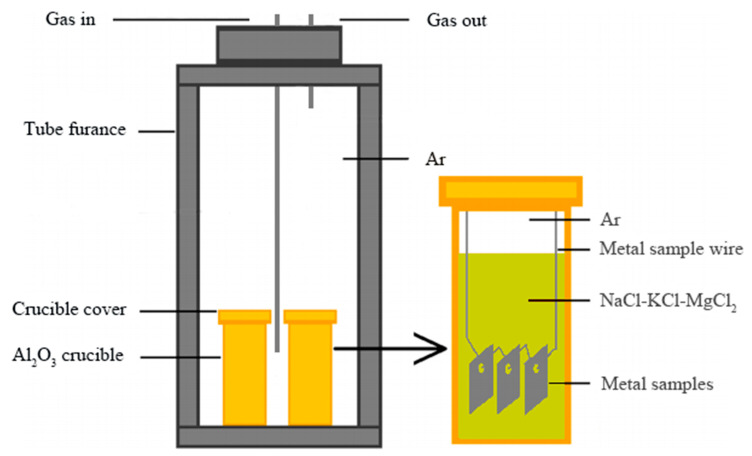
Schematic diagram of the static corrosion setup.

A custom-designed vertical tube furnace, as shown in Figure 1, was used in this experiment. The two groups of Al_2_O_3_ crucibles, filled with molten salt and specimens, were carefully transferred from the argon-filled glove box to the vertical tube furnace, minimizing exposure to air. The corrosion experiments were conducted at 700 °C for durations of 2, 5, 10, 20, 50, and 100 h. After the experiments, the furnace was allowed to cool to room temperature before removing the crucibles. The specimens, encased in molten salt, were carefully retrieved and cleaned with a specialized solution consisting of 15% HCl and 85% deionized water to remove surface corrosion residues. This step aimed to eliminate corrosion products that could interfere with subsequent analyses, ensuring the cleanliness of the specimen surfaces. For further removal of potential residues, the specimens were ultrasonically cleaned in deionized water and anhydrous ethanol at room temperature for about 10 min each. This ensured the removal of all potential contaminants, preventing contamination during characterization analyses [17].

After cleaning and drying, the mass of the three specimens in Group A was precisely measured on an electronic balance and recorded as *m*_1_, *m*_2_, and *m*_3_. This mass measurement not only facilitates the calculation of corrosion rates but also provides a quantitative basis for exploring the performance changes of materials under specific corrosion conditions.

The formula for calculating the mass loss over time is as follows [18]:

$$\frac{\Delta m}{S_{0}}=\frac{m_{0}-m_{x}}{S_{0}}.$$

Here, *m*_0_ represents the initial mass of the sample in grams (g), *m_x_* (*x* = 1, 2, 3) denotes the mass of the sample at time t in grams (g), and *S*_0_ is the initial surface area of the sample in square centimeters (cm^2^).

Finally, by calculating the average mass change rate of the three parallel specimens, the rate of mass change for alloys with varying Cr contents at corrosion time t can be obtained.

After undergoing the meticulous preprocessing procedure, the specimens in Group B were placed in an argon-filled glove box to ensure they were presented in a pure and uncontaminated state for subsequent characterization. These characterizations include microscopic structure and composition analysis, as well as studies on the corrosion mechanism, thereby utilizing advanced analytical techniques such as Scanning Electron Microscopy (SEM), Energy Dispersive Spectroscopy (EDS), and X-ray Diffraction (XRD) to obtain reliable experimental data.

This study also employs ICP-OES experiments to determine the solubility of Ni and Cr in NaCl-KCl-MgCl_2_ molten salt in Ni-Cr alloys.

### 2.3. Analytical Methods

The surface morphology and cross-sectional appearance of the samples post-corrosion were meticulously examined using a Scanning Electron Microscope (SEM). The samples were carefully prepared through standard grinding and polishing procedures for cross-sectional SEM and EDS analysis. To prevent damage to the corrosion products during sample preparation, the samples were encapsulated in an organic polymer matrix. The chemical composition of the corrosion products on the sample surface and the elemental loss in the cross-section were determined using an Energy Dispersive Spectrometer (EDS). The crystal structure of the corrosion products on the sample surfaces was characterized using X-ray Diffraction (XRD). Additionally, the dissolution of Ni and Cr from the alloy into the post-corrosion molten salt was quantitatively analyzed using Inductively Coupled Plasma Optical Emission Spectroscopy (ICP-OES).

In this study, we employed the EM-30AX+ Scanning Electron Microscope manufactured by COXEM (Daegu, Republic of Korea). This device is capable of visually presenting the microstructure of metal samples after corrosion, such as the size and distribution of corrosion pits, thus assessing the degree of corrosion. Additionally, its EDS functionality allows for the analysis.

The X-ray diffractometer used in this paper is the 18KW D/MAX2500V+/PC model manufactured by Rigaku Corporation in Tokyo, Japan. It utilizes a monochromatized Cu Kα radiation source with a wavelength of λ = 1.54 Å. By comparing the XRD patterns of the samples with standard PDF cards, accurate phase analysis can be performed to determine the lattice structure of the materials being tested.

The study employed the Thermo Fisher Scientific-manufactured iCAP 7400 model of Inductively Coupled Plasma Optical Emission Spectrometer (ICP-OES) (Waltham, MA, USA). It was used to analyze the elemental content of NaCl-KCl-MgCl_2_ salt before and after corrosion, aiming to predict the corrosion degree of the alloy.

This study employs ICP-OES experiments to determine the solubility of Ni and Cr in NaCl-KCl-MgCl_2_ molten salt in Ni-Cr alloys, aiming to better verify the corrosion rates of Ni-Cr alloys with different Cr contents in the molten salt, in comparison with mass change experiments. At the end of each experimental set, molten salt samples from group A after corrosion are collected, and a sample weight of 0.2867 g (±0.0001 g) is measured using an electronic balance. Subsequently, the sample is subjected to pretreatment in a microwave digestion vessel. HCl is utilized as the solvent to effectively dissolve the sample and release metal ions into the solution. The microwave digestion process is conducted using a digestion apparatus, heating the solution until the volume reduces to approximately 1 mL. Care is taken to observe the solution for the absence of obvious bubbles when heating is stopped, and complete drying is avoided to prevent the loss of volatile elements. After cooling, the solution is diluted to 10 mL with distilled water, ensuring the accuracy of dilution using a calibrated volumetric flask, and liquid is added until it reaches the mark. Consistency and accuracy are ensured with each operation. After dilution, the sample is thoroughly mixed for sufficient time using a mechanical shaker or manual agitation to ensure uniformity. A series of standard solutions of Ni and Cr are prepared, covering concentrations ranging from low to high, to establish a standard curve. Calibration of the ICP-OES is performed to ensure reading accuracy. The processed sample solutions are sequentially injected into the ICP-OES, and the spectral intensities of Ni and Cr in the samples are measured and recorded. Finally, the measured spectral intensities are converted into actual elemental concentrations using the standard curve, and the content of Ni and Cr in each sample is calculated.

## 3. Results

### 3.1. Mass Change Experiment

**Figure 2:**
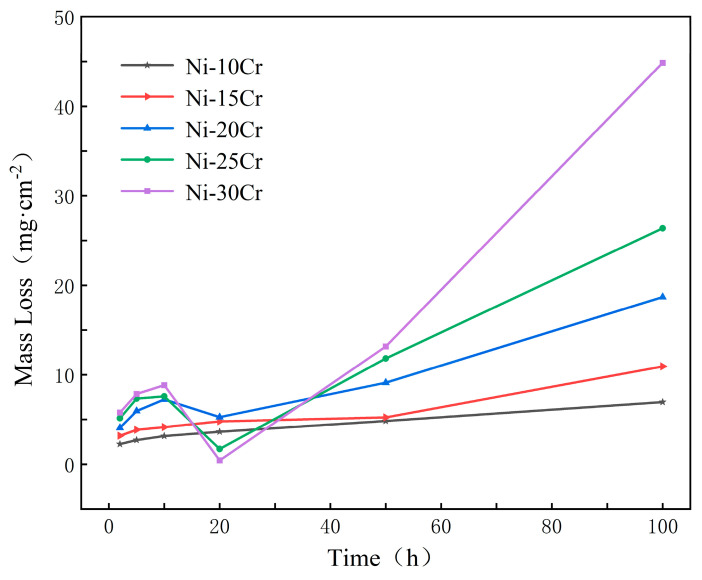
Mass change of Ni-Cr alloys with different Cr contents in NaCl-KCl-MgCl_2_ molten salt from 2 h to 100 h.

The mass loss of five Ni-Cr alloys with different Cr contents submerged in NaCl-KCl-MgCl_2_ molten salt at 700 °C for durations of 2 h, 5 h, 10 h, 20 h, 50 h, and 100 h is illustrated in Figure 2.

Figure 2 reveals that during the initial stages of corrosion (2 to 10 h), the mass loss of all alloys increases with the duration of exposure. In this phase, alloys with lower Cr content, such as Ni-10Cr and Ni-15Cr, exhibit lesser mass loss compared to those with higher Cr content, like Ni-30Cr. This trend might be attributed to the higher Cr content facilitating the formation of more corrosion products in the initial stages, leading to greater mass loss in the short term. By the 20 h mark, the situation becomes particularly interesting. For Ni-25Cr and Ni-30Cr alloys, a significant reduction in mass loss is observed, especially for the Ni-30Cr alloy, which shows almost no loss. This could indicate the formation of a protective oxide layer on these alloys in the corrosive environment, slowing down the corrosion process. However, the mass loss for Ni-10Cr, Ni-15Cr, and Ni-20Cr continues to increase steadily at 20 h. By 50 h, all alloys experience an increase in mass loss, notably for the Ni-30Cr alloy, suggesting that even the presence of a protective layer can result in its breakdown or other forms of accelerated corrosion over prolonged exposure. At 100 h, significant increases in mass loss are noted across all alloys, with the highest content alloy (Ni-30Cr) experiencing the greatest loss throughout the experiment, reaching 44.87 mg/cm². This indicates that over the long term, increased Cr content may lead to more corrosion product accumulation or breakdown of the oxide layer.

These data suggest that while an increase in Cr content may not reduce alloy mass loss in the initial stages, over a certain period, a higher Cr content can lead to the formation of a more stable protective layer on the alloy’s surface, thereby slowing the corrosion rate. However, over extended periods of corrosion, alloys with high Cr content do not appear to exhibit superior corrosion resistance. This underscores that for Ni-Cr alloys’ corrosion resistance, not only is the Cr content itself significant, but so too is the behavior of the alloy in specific corrosive environments. Further research is needed to optimize alloy compositions for superior corrosion resistance in molten salt environments.

### 3.2. ICP-OES

**Figure 3:**
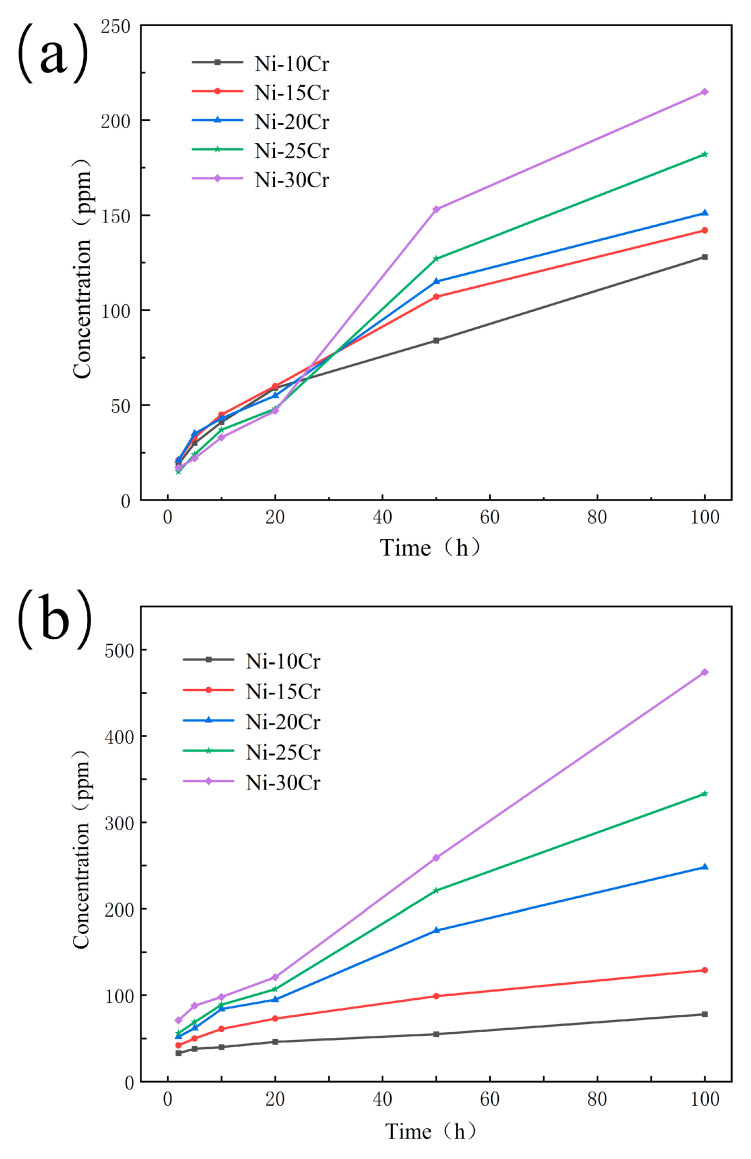
Concentrations of Ni and Cr in NaCl-KCl-MgCl_2_ molten salt after 2 to 100 h of corrosion for Ni-Cr alloys with different Cr contents. (**a**) represents Ni, and (**b**) represents Cr.

Figure 3 presents the dissolution quantities of Ni and Cr elements, measured in parts per million (ppm) through Inductively Coupled Plasma Optical Emission Spectrometry (ICP-OES), for five different Ni-Cr alloys corroded in NaCl-KCl-MgCl_2_ molten salt over various durations. By integrating the ICP-OES dissolution data with the mass loss figures, we can delineate the corrosion process into three distinct phases for a more nuanced analysis:

1. Short-term Corrosion (2 to 20 h)

In the initial phase, there is a rapid increase in the dissolution rates of Cr, particularly noticeable in the Ni-30Cr alloy. This suggests that despite the formation of an oxide layer on the alloy surface, this layer is not sufficiently stable in the early stages to effectively prevent chromium dissolution. Meanwhile, the dissolution rate of Ni also increases but at a comparatively slower pace. This indicates that Ni might be more stable during the initial stages of corrosion, or that chromium is more prone to dissolution. In this phase, the Ni-25Cr and Ni-30Cr alloys exhibit relatively lower mass loss, which could be attributed to the formation of an initial, somewhat protective oxide layer on the surface due to the higher Cr content [19].

2. Mid-term Corrosion (20 to 50 h)

A notable increase in Cr dissolution is observed across all alloys, especially in the Ni-30Cr alloy, indicating that the corrosion mechanism may have transitioned, such as through molten salt permeating the oxide layer or localized fracturing of the oxide layer. During this stage, a steady increase in Ni dissolution suggests ongoing corrosion attacks on the Ni matrix. The reduced mass loss observed in the Ni-25Cr and Ni-30Cr alloys at 20 h may be attributed to the formation of a more comprehensive oxide layer, which provides improved protection during the mid-term corrosion phase.

3. Long-term Corrosion (100 h)

Significant increases in Cr dissolution across all alloys, particularly those with high Cr content, align with the long-term trends in mass loss. This suggests that, despite the presence of oxide layers, prolonged corrosion leads to notable Cr dissolution. The Ni-30Cr alloy exhibits the highest Ni and Cr dissolution after extended corrosion, supporting the hypothesis that protective layers formed in high Cr content alloys may fail over time, accelerating corrosion. The trend of Ni dissolution is relatively stable but also significantly increases over the long term, indicating that Ni corrosion is a slow yet persistent process.

Considering the entire corrosion process, alloys with higher Cr content may initially form thicker oxide layers, but the stability of these layers in corrosive environments, especially under long-term corrosion conditions, warrants further investigation. The significant impact of Cr dissolution on alloy mass loss highlights the selective corrosion of Cr as a key factor. High Cr dissolution during prolonged corrosion may relate to degradation of the protective layer or other microstructural changes. In designing Ni-Cr alloys, enhancing initial corrosion resistance by increasing Cr content is important, but optimizing overall corrosion resistance to withstand long-term corrosive environments is equally crucial. This may involve adjusting other elements in the alloy or developing new alloy treatment techniques to enhance the stability of the oxide layer.

### 3.3. Surface SEM Analysis

**Figure 4:**
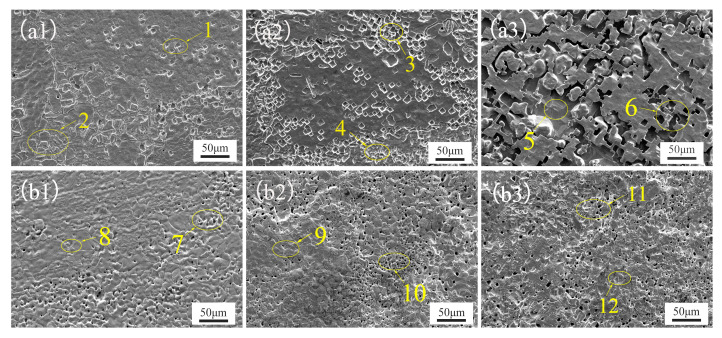
Scanning Electron Microscope (SEM) images of the surface of a low-Cr alloy after corrosion in a NaCl-KCl-MgCl_2_ molten salt. (**a1**–**a3**) show Ni-10Cr after 20 h, 50 h, and 100 h of corrosion, respectively, while (**b1**–**b3**) show Ni-15Cr after 20 h, 50 h, and 100 h of corrosion, respectively.

**Figure 5:**
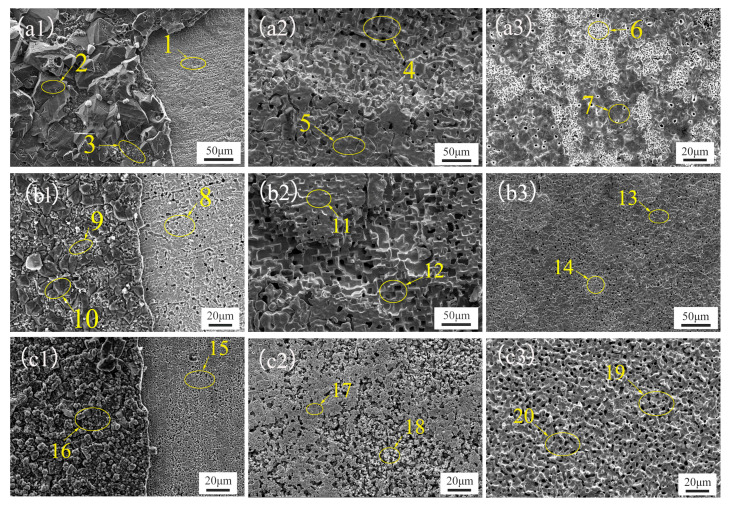
Scanning Electron Microscope (SEM) images of the surface of a high-Cr alloy after corrosion in a NaCl-KCl-MgCl_2_ molten salt. (**a1**–**a3**) show Ni-20Cr after 20 h, 50 h, and 100 h of corrosion, respectively, (**b1**–**b3**) show Ni-25Cr after 20 h, 50 h, and 100 h of corrosion, respectively. while (**c1**–**c3**) show Ni-30Cr after 20 h, 50 h, and 100 h of corrosion, respectively.

The surface scanning electron microscope (SEM) images of Ni-Cr alloys with five different Cr contents after 20 h, 50 h, and 100 h of corrosion in a NaCl-KCl-MgCl2 mixed salt at 700 °C are shown in Figure 4 and Figure 5. Specifically, Figure 4 presents the SEM images of the alloys with low Cr content (Ni-10Cr, Ni-15Cr), while Figure 5 shows those of the alloys with high Cr content. The elemental mass fractions obtained from the EDS analysis of the corresponding regions in Figure 4 and Figure 5 are listed in Table 3.

Figure 4(a1–a3) illustrate the surface morphology of Ni-10Cr after 20 h, 50 h, and 100 h of corrosion, respectively. After 20 h of exposure, the surface of the Ni-10Cr alloy exhibited fine corrosion pits and slight etching, characteristic of localized pitting corrosion typically observed at lower chromium contents. As the corrosion progressed to 50 h, both the depth and breadth of corrosion intensified, with pitting expanding to form larger cavities. The material at the edges might begin to flake off due to the failure of local protective layers, indicating signs of intergranular corrosion. With further corrosion time extended to 100 h, the corrosion pits and etching on the surface became more pronounced, suggesting an advancing corrosion process, potentially transitioning to more concentrated pitting and intergranular corrosion, especially at internal and interfacial areas of the material.

For Ni-15Cr, Figure 4(b1–b3) reveal surface corrosion phenomena after 20 h of exposure, characterized by numerous tiny corrosion pits. These small yet numerous pits indicate that even an early stage of corrosion could not be completely prevented by the increased Cr content. After 50 h of corrosion, the number of pits significantly increased and were more densely distributed. Although the size of individual pits remained relatively small, the corrosion had covered a more extensive area overall. This reflects that despite the protective role of increased Cr content, the corrosion process continued unabated, predominantly in the form of micropitting. By 100 h, the number of pits on the alloy surface further increased, with some beginning to merge into larger corroded areas. Although these pits were still relatively smaller compared to those on Ni-10Cr alloy, their sheer number was significantly higher than that of Ni-10Cr. This may imply that although a higher Cr content was present, the effectiveness of the corrosion protection layer was limited over the long term, failing to prevent the occurrence and development of corrosion. In summary, both low-Cr alloys exhibited the formation of pits at the initial stage of corrosion, but as time progressed, the pits on Ni-10Cr alloy became larger and deeper, whereas Ni-15Cr alloy showed a higher number of finer pits. This could reflect the impact of Cr content on the corrosion morphology, where a higher Cr content, although unable to prevent pit formation, appeared to slow down the pit enlargement process. This suggests that chromium plays a role in inhibiting the expansion of corrosion pits; however, the number of pits continued to increase. These fine pits could converge into larger corroded areas over a longer corrosion duration, leading to an overall elevation in the level of corrosion.

Table 3 displays the elemental distribution in the corroded areas identified in the SEM images, and the analysis of EDS data and SEM imagery reveals the selective corrosion characteristics of Cr and its progression over time in the Ni-10Cr and Ni-15Cr alloys immersed in a NaCl-KCl-MgCl_2_ molten salt. For the Ni-10Cr alloy, after 20 h of corrosion, the relatively high content of Cr suggests that a protective layer of Cr might have formed on the alloy surface under the initial influence of the molten salt. After 50 h, the Cr content begins to decrease in certain areas, likely due to Cr experiencing a more rapid dissolution process in these regions. The Cr oxide layer is compromised, exposing the underlying Ni matrix. After 100 h, the Cr content in some areas drops to zero, indicating that Cr has completely corroded away from these areas, leaving behind regions enriched in Ni. These areas exhibit larger corrosion pits and more extensive etching in the SEM images, indicating a progression to more severe corrosion as the protective Cr layer is breached and depleted.

For the Ni-15Cr alloy, after 20 h of corrosion, the Cr content remains high, yet compared to the Ni-10Cr alloy, a greater number of small corrosion pits begin to appear. This indicates that even with a higher Cr content, selective corrosion cannot be completely prevented. As corrosion time extends to 50 and 100 h, the detected Cr content in the corroded areas continues to decrease. This decline in Cr content corresponds with an increase in corrosion pits, and the surface morphology indicates that corrosion is still progressing. Unlike the Ni-10Cr alloy, despite having more chromium, the anticipated stronger protective effect was not observed. Instead, the corrosion appears to be more dispersed and widespread. After 100 h, although chromium has not entirely vanished, the ubiquity of corrosion and the distribution of pits suggest that the effectiveness of the chromium protective layer in resisting ongoing corrosion is diminishing.

These observations might suggest that while theoretically, increasing chromium content should offer better protection, in these alloys, corrosion instead intensified with increased chromium content. A possible explanation is that the increased chromium content makes the alloy more susceptible to selective corrosion, especially in areas where a complete protective layer has not formed, or where the protective layer has been breached for some reason, leading to faster chromium depletion. Another potential factor is that, although chromium forms protective oxide layers, these layers may not be stable enough under the action of the corrosive medium, or regions of chromium enrichment in the alloy may become preferred points of attack for corrosion, also leading to faster overall chromium loss and thereby exacerbating corrosion.

From this analysis, it is evident that as the Cr content increases from 10 wt% to 15 wt%, the corrosion behavior of Ni-Cr alloys in a NaCl-KCl-MgCl_2_ molten salt exhibits a non-intuitive pattern, where the degree of corrosion does not decrease noticeably with higher Cr content, and in some instances, appears to worsen. To further clarify the relationship between Cr content and corrosion behavior, as well as to explore the optimal balance of Cr content in alloy design, it becomes necessary to investigate the performance of Ni-Cr alloys with higher Cr contents under the same corrosive conditions.

Figure 5 showcases the surface scanning electron microscope (SEM) photographs of high-Cr-content Ni-Cr binary alloys (Ni-20Cr, Ni-25Cr, Ni-30Cr) after corrosion in a NaCl-KCl-MgCl_2_ molten salt. Table 4 presents the results of the energy-dispersive spectroscopy (EDS) point scans conducted on the surfaces of the three alloys following the corrosion tests. Through the analysis of SEM images and EDS data, an assessment can be made regarding the behavior of these three high-Cr-content Ni-Cr alloys in the NaCl-KCl-MgCl_2_ molten salt environment over varying corrosion durations.

For these three alloys, SEM images taken after 20 h show the formation of a distinct protective film in certain areas, while EDS data reveal that these films primarily comprise Mg, Cr, and O. This indicates that in the molten salt environment, Cr and Mg may react with oxygen to form a protective oxide layer on the alloy surface. However, in areas where the protective layer has not formed, the alloys still suffer significant corrosion, suggesting that the formation of the protective layer might be uneven. This unevenness could lead to concentrated attacks by the corrosive medium in unprotected areas, exacerbating local corrosion phenomena.

As corrosion progresses to 50 h, the protective films observed at 20 h are no longer evident in the SEM images, possibly indicating that the protective layers have been compromised and eroded by the molten salt. The disappearance of the protective films could be due to the insufficient stability of Cr and Mg oxides to withstand the long-term high-temperature molten salt environment, allowing the corrosion process to penetrate through the protective layer and continue corroding the underlying alloy. The alloy surfaces become rougher and more irregular, and the shape and distribution of pits suggest that the initially uniform corrosion has begun to shift towards localized and concentrated corrosion. This shift could be a result of the corrosive medium directly reacting with the alloy substrate following the rupture of the protective layer. This type of corrosion may include pitting, as deeper corrosion pits are observed in localized areas. On the other hand, some smoother areas might still retain remnants of the protective layer, exhibiting less severe corrosion. This indicates that the presence of the protective layer has a certain effectiveness in resisting corrosion.

In our investigation into the corrosion behavior of low-Cr-content Ni-Cr alloys, we encountered an intriguing phenomenon: contrary to expectations, increasing the Cr content did not decelerate the corrosion process but instead led to a higher number of corrosion pits. This trend of worsening corrosion morphology with added Cr content was also confirmed in alloys with higher Cr concentrations. Through SEM imaging and EDS analysis, it was observed that alloys with higher Cr content, such as Ni-20Cr, Ni-25Cr, and Ni-30Cr, exhibited a significant increase in the number of corrosion pits, suggesting that an increase in Cr content might have, to some extent, facilitated the formation of new corrosion sites. This discovery underscores an important concept: in Ni-Cr alloys, merely increasing the chromium content does not necessarily offer better corrosion protection. On the contrary, an excess of chromium might become a catalyst in the corrosion process due to its selective corrosion in the corrosive environment. This could be related to the heterogeneity of chromium-rich areas or to the quality and stability of chromium oxide protective layers. In high-temperature molten salt corrosion environments, these protective layers might become more susceptible to damage, thereby manifesting as exacerbated corrosion at the macroscopic level.

### 3.4. Cross-Sectional SEM and EDS Analysis

**Figure 6:**
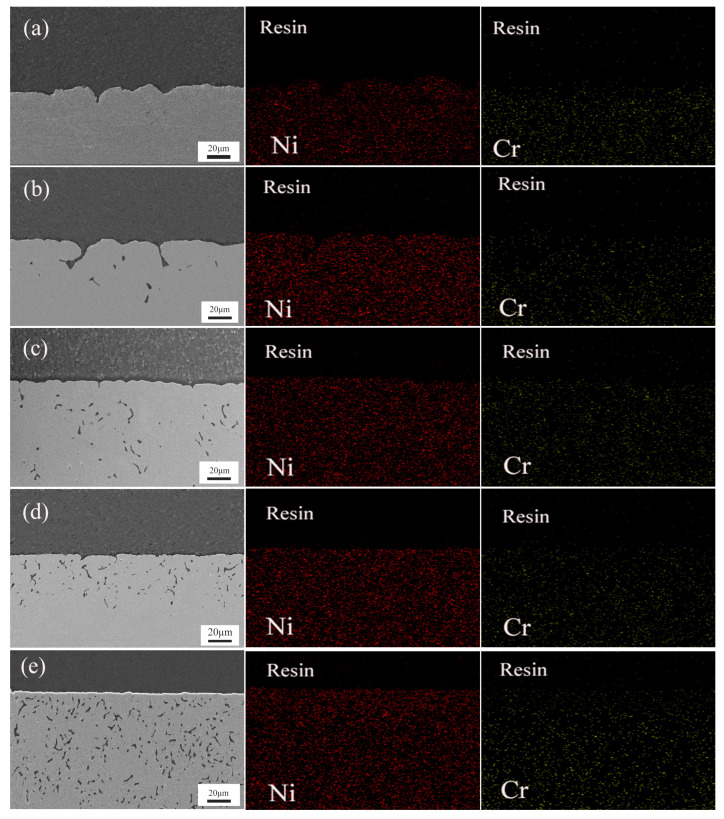
Cross-sectional Scanning Electron Microscope (SEM) images and corresponding EDS surface scans of Ni-Cr alloys with varying Cr contents after corrosion in NaCl-KCl-MgCl_2_ molten salt. (**a**) represents Ni-10Cr, (**b**) represents Ni-15Cr, (**c**) represents Ni-20Cr, (**d**) represents Ni-25Cr, and (**e**) represents Ni-30Cr.

Figure 6 presents the SEM images of the cross-sections of Ni-Cr binary alloy blocks with varying Cr contents after 100 h of corrosion in a NaCl-KCl-MgCl_2_ molten salt at 700 °C, accompanied by their EDS surface scanning elemental analysis. When analyzing the EDS results of the Ni-Cr alloys with different Cr contents, we noticed the presence of Cr-depleted regions across all alloys. This indicates that the Cr elements in these areas were preferentially dissolved during the corrosion process, serving as direct evidence of selective Cr corrosion. The SEM image of the Ni-15Cr alloy notably reveals the formation of holes due to corrosion, which are the result of the corrosive medium penetrating through the protective layer and eroding the substrate.

Figure 6a displays the SEM image of the Ni-10Cr alloy’s cross-section, which appears comparatively uniform with mild corrosion. The EDS analysis shows an even distribution of Ni and Cr, indicating that at lower Cr contents, the impact of corrosion is limited. As the Cr content increases, Figure 6b reveals more pronounced corrosion in the SEM image, with the emergence of holes suggesting that corrosion can penetrate the initial protective layer and begin to impact the alloy’s substrate even at relatively lower Cr contents. The EDS results highlight a significant reduction in Cr content as corrosion progresses, especially near corrosion pits, making the Cr-depleted regions more pronounced [20]. Figure 6c–e illustrate that with an increase in Cr content, the number of holes in the SEM images significantly multiplies, and surface corrosion becomes more evident, indicating more severe localized corrosion phenomena. In the Ni-20Cr, Ni-25Cr, and Ni-30Cr alloys, the increase in Cr-depleted regions as shown in the EDS results correlates positively with the increase in the number of holes, suggesting a direct link between Cr depletion and the degree of alloy corrosion. In alloys with higher Cr contents, the proliferation of Cr-depleted areas implies that, despite a higher overall Cr content, once Cr-depleted regions form, they become susceptible points for corrosion. The selective corrosion of Cr leaves the remaining Ni matrix more vulnerable to erosion by the corrosive medium.

The analysis results from the cross-sectional SEM images and EDS data are consistent with conclusions drawn from mass loss experiments and observations from surface SEM and EDS: as the Cr content in Ni-Cr alloys increases, the severity of corrosion actually escalates. Cross-sectional SEM analysis reveals that with higher Cr content, the internal structure of the alloys becomes increasingly unstable, and porosity increases, indicating more pronounced internal corrosion phenomena. This is further corroborated by EDS analysis, showing that high-Cr-content alloys have more extensive Cr-depleted areas, directly correlating with increased corrosion severity. Mass loss experiments reveal greater mass loss in high-Cr-content alloys with prolonged corrosion time. These experimental outcomes correspond with observations of selective Cr corrosion and increased pore formation from cross-sectional SEM and EDS analyses. Observations from surface SEM and EDS also support this finding, revealing more porosity and corrosion pits on the alloy surfaces as Cr content increases.

This series of analyses highlights a critical issue: in specific corrosive environments, simply increasing the Cr content in an alloy does not necessarily enhance its corrosion resistance. On the contrary, a high Cr content might lead to exacerbated corrosion due to increased selective Cr corrosion. This could be related to the microscale heterogeneity of high-Cr-content alloys, which form Cr-depleted regions vulnerable to attack in corrosive environments, thereby becoming hotspots for accelerated corrosion.

Therefore, when designing Ni-Cr corrosion-resistant alloys for use in high-temperature molten salt environments, considerations should extend beyond Cr content to include the uniformity of Cr distribution, the microstructure of the alloy, and other factors that may affect corrosion behavior. These findings provide important guidance for optimizing alloy corrosion resistance, pointing towards directions for future research, especially in the development of high-performance corrosion-resistant materials.

### 3.5. X-ray Diffraction Analysis

**Figure 7:**
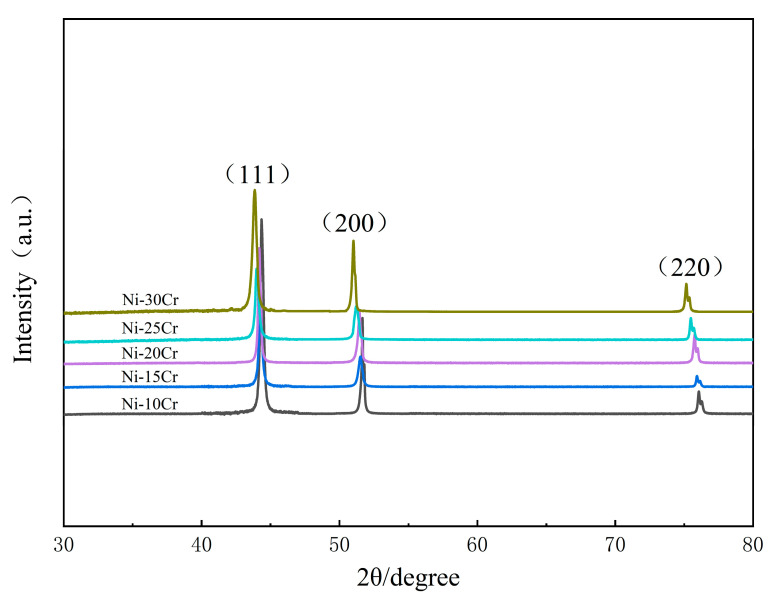
XRD patterns of Ni-Cr binary model alloys with different compositions before corrosion.

Figure 7 presents the XRD patterns of Ni-Cr binary model alloys with different ratios. It can be observed from the figure that alloys with a Cr content of less than 30 wt.% all fall within the gamma (γ) solid solution phase of nickel, with no new precipitate phases appearing during the processes of melting, rolling, or high-temperature annealing [21]. As illustrated in Figure 7, as the Cr content in the model alloys increases, the peak positions gradually shift to the left. According to Bragg’s law, this leftward shift of peak positions is primarily due to an increase in the lattice constant. Bragg’s law is as follows:

2*d*·sin *θ* = *nλ*.

In the formula, *d* represents the interplanar spacing, *θ* is the angle between the incident X-rays and the crystal plane, *n* is the diffraction order, *λ* is the wavelength of the incident X-rays, and it is a constant. The formula indicates that when the peak positions shift to the left, indicating a decrease in *θ*, the value of *d* increases, meaning the lattice constant becomes larger. According to the periodic table, the atomic radius of Cr is larger than that of Ni. When Cr and Ni form a substitutional solid solution, it causes lattice distortion in Ni. As the Cr content in the alloy increases, the lattice distortion of Ni gradually intensifies, leading to an increase in the lattice constant.

**Figure 8:**
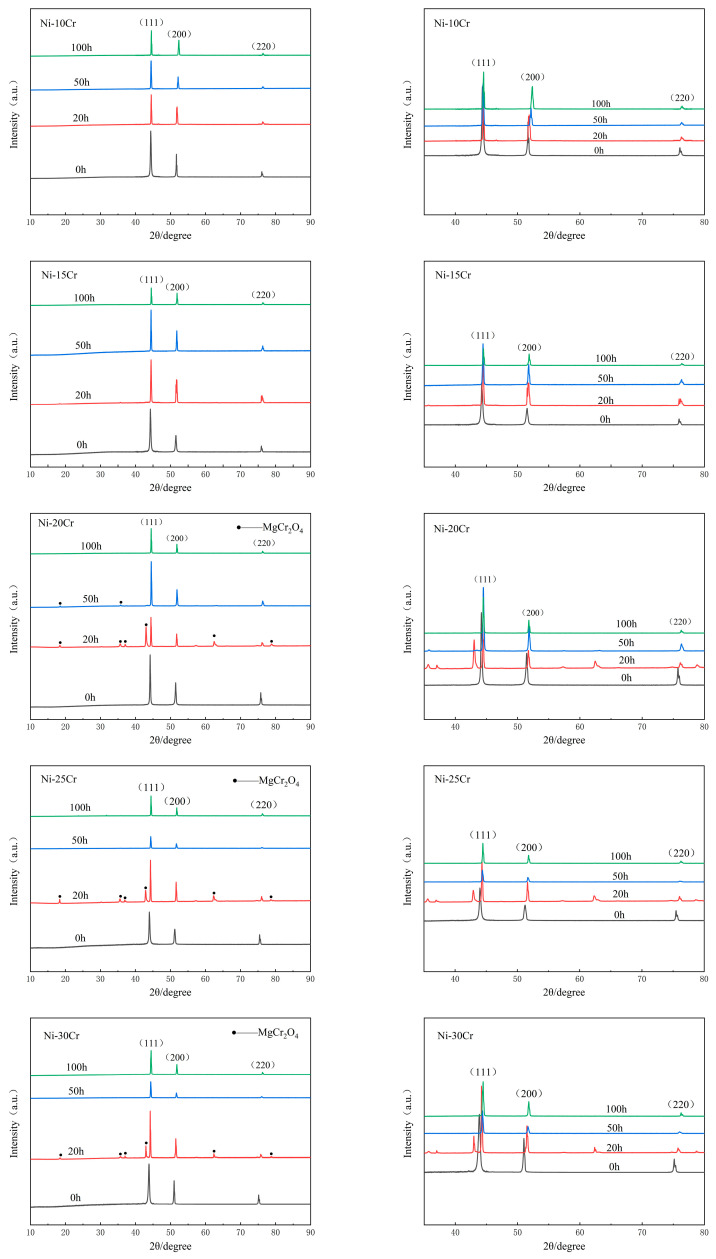
X-ray diffraction images of Ni-Cr binary alloys with different Cr contents before corrosion and after corrosion for 20 h, 50 h, and 100 h.

Figure 8 shows the XRD patterns of binary Ni-Cr alloys after corrosion. In the patterns for low-Cr alloys (Ni-10Cr, Ni-15Cr), apart from the three strong peaks of Ni, no new peaks appear, indicating the absence of insoluble corrosion products adhering to the alloy surface after corrosion. For high-Cr alloys (Ni-20Cr, Ni-25Cr, Ni-30Cr), in addition to the three strong Ni peaks, clear peaks of magnesiochromite (MgCr_2_O_4_) can be observed in the spectra after 20 h of corrosion. This suggests that the protective film composed of Mg, Cr, and O observed on the surface of high-Cr alloys in previous SEM and EDS analyses is chemically MgCr_2_O_4_. With the corrosion duration extending to 50 and 100 h, except for the Ni-20Cr alloy, which still shows residual MgCr_2_O_4_ peaks after 50 h of corrosion, the surfaces of other high-Cr alloys do not display MgCr_2_O_4_ peaks after 50 and 100 h of corrosion. This indicates that MgCr_2_O_4_ formed on the surface of high-Cr alloys also gradually corrodes over time, which is consistent with the SEM and EDS analysis results.

Comparative analysis of the peak positions in the XRD patterns of different ratios of Ni-Cr alloys after varying corrosion durations, as seen in Figure 8, reveals that the three strong peaks of Ni-Cr alloys gradually shift to the right with the prolongation of corrosion time. This peak shift further corroborates the selective corrosion behavior of Cr. The corrosion of Cr on the alloy surface leads to the formation of vacancies, and during the high-temperature corrosion process, the accumulation of vacancies in the Ni-Cr alloy causes the gradual formation of a pure Ni lattice structure in the corroded areas of the alloy surface.

Compared to Figure 7, the X-ray diffraction (XRD) peaks of Ni-Cr binary alloys with different compositions after corrosion exhibit differences, yet they show a converging trend. Calculations based on Bragg’s equation indicate that, before corrosion, the interplanar spacings for the (110), (200), and (220) crystal planes of Ni-Cr binary alloys were within the ranges of 2.0409 < *d* < 2.0572, 1.7676 < *d* < 1.7814, and 1.2568 < *d* < 1.2623, respectively. After 100 h of corrosion, these ranges narrowed to 2.0348 < d < 2.0366, 1.7622 < *d* < 1.7629, and 1.2465 < *d* < 1.2473, respectively, indicating that the alloy’s interplanar spacings began to approach those of pure nickel (Ni). The interplanar spacings of the (110), (200), and (220) crystal planes for pure Ni are *d* = 2.0344, *d* = 1.7619, and *d* = 1.2458 (PDF#04-003-1538), respectively, showing that the crystal lattice structure of the Ni-Cr alloys after corrosion tends toward the lattice parameters of pure Ni.

This change reveals the significant impact of the corrosion process on the crystal structure of Ni-Cr binary alloys, particularly how the loss of Cr elements causes the alloy lattice to gradually shift toward the structure of pure Ni. Such alterations in lattice parameters offer an important perspective for understanding the behavior of Ni-Cr alloys in corrosive environments. This also highlights the crucial influence of alloy composition on its corrosion resistance properties.

## 4. Discussion

The corrosion mechanism of Ni-Cr alloys is a complex process, involving the interplay of various factors and reactions. Mass loss experiments and ICP-OES results have revealed that in Ni-Cr alloys with Cr contents ranging from 10% to 30%, the corrosion rate increases as the Cr content increases. By integrating the analysis results from SEM, EDS, and XRD, we can gain a deeper understanding of the corrosion mechanisms of these alloys in a NaCl-KCl-MgCl_2_ molten salt environment.

SEM analysis reveals that with an increase in Cr content, the number of surface pores in the alloys increases, especially in Ni-25Cr and Ni-30Cr alloys, indicating a heightened degree of corrosion. These pores reflect changes in the material’s internal microstructure and denote the localized nature of the corrosion reaction [22]. EDS analysis further unveils the presence of Cr-depleted regions across all alloys, particularly more extensive in alloys with higher Cr content. The emergence of these regions indicates that Cr elements are selectively dissolved during the corrosion process, reducing the overall corrosion resistance of the alloy.

Moreover, XRD analysis provides valuable insights into the phase changes occurring in the alloys during the corrosion process. The reduction in Cr content and the appearance of protective oxides formed by Cr, such as MgCr_2_O_4_, suggest that Cr is being consumed and corrosion products are forming on the surface and inside the alloy. Especially in high-Cr-content alloys, XRD exhibits more peaks for Cr oxides, consistent with the increased degree of corrosion.

MgCl_2_, due to its significant hygroscopic nature, readily absorbs moisture from the environment to form magnesium chloride with crystalline water [16], even though the molten salt used in this experiment was treated at high temperatures for purification, it’s challenging to completely remove moisture, and the risk of reabsorption of water remains. Under high temperatures, magnesium chloride with crystalline water (MgCl_2_·6H_2_O) undergoes a dehydration reaction, ultimately forming magnesium oxide (MgO), accompanied by the release of water and possibly generating other by-products such as hydrogen chloride (HCl). The chemical reaction equation is as follows:

MgCl_2_·6H_2_O = MgO + 2HCl (g) + 5H_2_O (g). (1)

In a NaCl-KCl-MgCl_2_ molten salt mixture, NaCl and KCl are chloride salts that, when melted at high temperatures, dissociate into their respective positive and negative ions, as does MgCl_2_. The chlorine elements in these compounds exist in the form of Cl^−^ ions.

Specifically, when these salts melt, the following dissociation reactions occur:

NaCl → Na^+^ + Cl^−^, (2)

KCl → K^+^ + Cl^−^, (3)

MgCl_2_ → Mg^2+^ + 2Cl^−^. (4)

These reactions produce free chloride ions, which are highly reactive within the molten salt, capable of migration and participation in processes such as corrosive reactions with metal oxides [23]. Under high-temperature conditions, these chloride ions exhibit significant chemical activity, allowing them to react with metals and their oxide layers, thereby facilitating the corrosion process. Given that molten salts typically operate at high temperatures, this further amplifies the corrosive potential of chloride ions.

The Gibbs free energy of formation for divalent alloy element chlorides increases in the order of Cr < Ni [24], a trend that corresponds to the relative stability of these metal elements in molten chloride salts. Hence, it can be concluded that since CrCl_2_ has the highest negative Gibbs free energy of formation, Cr will be preferentially attacked. Research by Zahs and Kawahara lso indicates that Cr is preferentially attacked in a chloride atmosphere, followed by Ni [25,26]. Based on the above analysis, the primary corrosion reaction of alloys in a NaCl-KCl-MgCl_2_ melt can be inferred as follows: Cr preferentially dissolves into the molten salt, forming soluble CrCl_2_.

Cr + 2Cl^−^ = CrCl_2._ (5)

Subsequently, CrCl_2_ in the molten salt is further oxidized to CrCl_3_, with the chemical reaction equation as follows:

CrCl_2_ + Cl^−^ = CrCl_3._ (6)

It should be noted that chromium oxides are stable in molten chloride salts, as previously reported in several studies on the corrosion of alloys in molten chloride salts in the air [27]. Therefore, Cr may react directly with O_2_ and H_2_O to form Cr_2_O_3_ on the alloy surface.

2Cr + 3H_2_O (g) = Cr_2_O_3_ + 3H_2_ (g)    ΔG = −295.5 KJ/mol, (7)

4Cr + 3O_2_ (g) = 2Cr_2_O_3_  ΔG = −878.1 KJ/mol. (8)

However, based on SEM and EDS results, neither Cr_2_O_3_ nor chromium-containing oxides were observed in the corrosion areas on or beneath the surface of low-Cr alloys. This suggests that under our experimental conditions, the Cr_2_O_3_ formed from the reaction of low-Cr alloys would ultimately be dissolved into the salt as CrCl_3_ by Cl^−^, even if it could form during the corrosion process.

Cr_2_O_3_ + 6Cl^−^ = 2CrCl_3_ + 3O^2−^. (9)

These findings indicate that alloys selectively dissolve Cr in the molten salt, forming soluble Cr ions, which leads to the formation of subsurface cavities and weight loss. Studies by Hosoya and Cho also show that in the presence of moisture, Cr in Ni-Cr alloys is selectively chlorinated under inert atmospheres, forming chlorides that enter the molten salt [28,29].

In low-Cr alloys, the protective layer formed by Cr_2_O_3_ is relatively thin and insufficient to block the erosion by chloride ions under long-term or high-temperature conditions. Chloride ions can penetrate or disrupt this thin oxide layer, leading to corrosion of the alloy substrate. Alloys with high Cr content may form a thicker and more continuous Cr_2_O_3_ layer on the surface. This denser oxide layer may offer greater resistance to the penetration of chloride ions, slowing down the corrosive action of chloride ions on Cr_2_O_3_.

At the same time, the good adhesion between the Ni matrix and Cr_2_O_3_ in Ni-Cr binary alloys is a key factor in providing effective protection. The presence of Ni facilitates the formation of a robust interface, which helps prevent the peeling or cracking of the Cr_2_O_3_ layer, especially under the influence of temperature changes or mechanical stress. This strong adhesion ensures the integrity and durability of the protective layer. The Ni matrix, being the main component of the alloy and occupying a significant volume, provides a stable oxidizing environment for the formation of a uniform and continuous Cr_2_O_3_ protective layer on its surface. Such a continuous oxide layer can effectively block further penetration of corrosive media, such as chloride ions.

In Ni-Cr alloys, the Ni matrix, which occupies a large volume, along with a higher Cr source in high-Cr alloys, provides protection for the Cr_2_O_3_ formed on the surface of high-Cr alloys, preventing it from being completely corroded by Cl^−^. The Cr_2_O_3_ that remains uncorroded reacts with MgO, which is produced by the hydrolysis of MgCl_2_·6H_2_O, to form MgCr_2_O_4_ that adheres to the alloy surface. The specific chemical reaction equation is as follows:

Cr_2_O_3_ + MgO = MgCr_2_O_4_  ΔG = −33.2 KJ/mol. (10)

It should be noted that these reaction equations are primarily based on theoretical conjectures and the chemical processes that may occur in a molten salt environment. The actual reactions and the specific mechanism of the disappearance of MgCr_2_O_4_ may be influenced by many factors, including the specific chemical composition of the molten salt, temperature, redox conditions, and other present chemicals.

In this experiment, the chemical reaction equations involving Gibbs free energy were calculated using HSC Chemistry 6.0 software, with the reaction temperature set at 700 °C.

## 5. Conclusions

This study primarily investigates the corrosion behavior of Ni-Cr binary alloys with different Cr contents in a molten salt environment. The corrosion characteristics of the alloys were analyzed in detail through mass loss experiments, ICP-OES, SEM, EDS, and XRD methods, and the corrosion mechanisms were discussed. The results of the study indicate:

1. The corrosion resistance of the alloys to the molten salt is closely related to the Cr content. As the Cr content increases, the degree of corrosion also increases. When the Cr content exceeds 15wt.%, the corrosion rate significantly increases, and the alloy surface exhibits noticeable corrosion pits merging and cracks propagating along grain boundaries, demonstrating characteristics of pitting and intergranular corrosion.

2. With the increase in Cr content, although the size of individual corrosion pits decreases, their number increases, suggesting that a higher Cr content may promote the formation of new corrosion sites.

3. In low Cr content alloys (Ni-10Cr, Ni-15Cr), no corrosion products were observed adhering to the alloy surface after corrosion. However, in high Cr content alloys (Ni-20Cr, Ni-25Cr, Ni-30Cr), after a 20 h corrosion experiment, a protective layer of MgCr_2_O_4_ formed on some surface areas, which could somewhat hinder the further progression of corrosion. Nevertheless, this protective layer was gradually consumed by corrosion as the exposure time extended to 50 h and 100 h.

## Figures and Tables

**Table 1:** The main physical and chemical properties of NaKMgCl salt at 700 °C.

Melting Point (°C)	Density (g/cm^3^)	Heat Capacity (J/g°C)	Viscosity (cP)	Thermal Conductivity (W/mK)
383	1.77	1.0238	6.4	0.47

**Table 2:** Chemical Composition (wt%) of Ni-xCr Alloy Samples.

Sample Number	Sample Name	Cr Content (wt%)
1	Ni-10Cr	10 ± 0.1
2	Ni-15Cr	15 ± 0.1
3	Ni-20Cr	20 ± 0.1
4	Ni-25Cr	25 ± 0.1
5	Ni-30Cr	30 ± 0.1

**Table 3:** Results of Energy Dispersive Spectroscopy (EDS) point scan analysis on the surfaces of two low-Cr alloys after corrosion testing.

Alloy	Corrosion Time (h)	Region	Ni (wt%)	Cr (wt%)
Ni-10Cr	20	1	94.4	5.6
	20	2	93.9	6.1
	50	3	91.1	8.9
	50	4	96.3	3.7
	100	5	100	0
	100	6	98.2	1.8
Ni-15Cr	20	7	89.3	10.7
	20	8	88.6	11.4
	50	9	94.8	5.2
	50	10	96.3	3.7
	100	11	99.2	0.8
	100	12	98.1	1.9

**Table 4:** Results of Energy Dispersive Spectroscopy (EDS) point scan analysis on the surfaces of three high-Cr alloys after corrosion testing.

Alloy	Corrosion Time (h)	Area	Ni (wt%)	Cr (wt%)	Mg (wt%)	O (wt%)
Ni-20Cr	20	1	91.7	8.3	…	…
	20	2	…	8.5	58.3	33.2
	20	3	…	12.3	54.9	32.8
	50	4	99.2	0.8	…	…
	50	5	98.3	1.7	…	…
	100	6	100	0	…	…
	100	7	98.1	1.9	…	…
Ni-25Cr	20	8	85.8	14.2	…	…
	20	9	…	43.1	30.1	26.8
	20	10	…	11.3	57.9	30.7
	50	11	99.8	1.2	…	…
	50	12	99.3	0.7	…	…
	100	13	99.3	0.7	…	…
	100	14	96.8	3.2	…	…
Ni-30Cr	20	15	77.8	22.2	…	…
	20	16	…	23.4	49.2	27.4
	50	17	81.6	18.4	…	…
	50	18	80.8	19.2	…	…
	100	19	94.1	5.9	…	…
	100	20	95.2	4.8	…	…

## Data Availability

The data presented in this study are available on request from the corresponding author. The data are not publicly available due to privacy concerns.
